# Molecular and physiological mechanisms of aging are distinct in the cardiac right and left ventricles

**DOI:** 10.1111/acel.14339

**Published:** 2024-09-19

**Authors:** Benjamin D. McNair, Aykhan Yusifov, Joshua P. Thornburg, Caleb R. Hoopes, Sushumna B. Satyanarayana, Tathagato Roy, Jason P. Gigley, Danielle R. Bruns

**Affiliations:** ^1^ Kinesiology and Health University of Wyoming Laramie Wyoming USA; ^2^ WWAMI Medical Education University of Washington School of Medicine Seattle Washington USA; ^3^ Molecular Biology University of Wyoming Laramie Wyoming USA

**Keywords:** cardiac aging, left ventricle, right ventricle, RNAseq

## Abstract

Aging is the primary risk factor for heart disease, the leading global cause of death. Right ventricular (RV) function predicts survival in several age‐related clinical contexts, yet no therapies directly improve RV function, in large part due to a poor mechanistic understanding of RV aging and how it is distinct from the widely studied left ventricle (LV). To address this gap, we comprehensively quantified RV functional and morphological remodeling with age. We further aimed to identify molecular mechanisms of RV aging thus we performed RNAseq on RV and LV from male and female young (4 months) and aged (19–21 months) C57BL6 mice. Contrary to the concentric hypertrophic remodeling and diastolic dysfunction that occurs in the LV, the aging RV underwent eccentric remodeling with significant dilation and impaired systolic function. Transcriptomic data were also consistent with ventricle‐specific aging, with few genes (13%) similarly shared between ventricles with aging. KEGG analysis identified shared aging genes in inflammatory and immune cell pathways that were confirmed by flow cytometry that demonstrated higher percent of GR1+ myeloid cells in both ventricles. Unique RV aging genes enriched in the biosynthesis of saturated fatty acids, PPAR signaling, and butanoate metabolism, and we identified putative novel RV‐specific aging genes. Together, we suggest that the RV and LV are unique cardiac chambers that undergo distinct remodeling with age. These robust differences may explain why therapies designed from LV‐based studies fail to improve RV function and suggest that future efforts emphasizing ventricular differences may elucidate new therapies for healthy cardiac aging.

## Graphical abstract

The right ventricle (RV) undergoes profound dilation with advanced age, concomitant with impaired systolic function. This RV‐specific pathophysiological phenotype occurs alongside RV‐specific transcriptomic changes with enrichment of lipid metabolism genes. Shared mechanisms of aging in both ventricles are likely due to cardiac inflammaging by upregulation of inflammatory pathways and GR1+ myeloid cells.

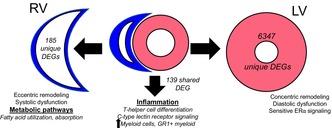

**Abbreviations**
DEG: differentially expressed genes
ERa: estrogen receptor alpha
LV: left ventricle
RV: right ventricle

## 1 INTRODUCTION

Cardiovascular disease is an age‐dependent condition, with more than 80% of deaths occurring in patients over the age of 65 (Ahmed, 2007). While commonly thought of as a disease of the left ventricle (LV), survival of patients with many forms of cardiovascular and pulmonary disease is dictated by *right ventricle* (RV) function (Goliasch et al., 2015; Haddad et al., 2008). That is, in age‐related disease, RV function predicts survival. However, despite the strong links between RV function and patient outcomes, virtually nothing is known about the aging RV and no RV‐directed therapies exist. Indeed, many therapies approved for LV dysfunction (i.e., beta‐blockers and inhibitors of the renin‐angiotensin‐aldosterone system) do not successfully treat RV failure (Borgdorff et al., 2013) and in some cases are even contraindicated (Prisco et al., 2020), further highlighting the need for RV‐centric investigations. An NHLBI working group best summarized the significance of studying the RV by stating that … “the RV cannot be understood simply by extrapolating data and experience from [the] LV …The RV is different from the LV” (Borgdorff et al., 2015). If meaningful progress to treat aged‐related cardiac disease is to be made, the RV must be studied and treated separately from the LV.

LV aging is characterized by myocyte and ventricular hypertrophy, resulting in impaired diastolic function and largely preserved systolic function at rest (Lakatta & Levy, 2003). These anatomical and physiological changes result in a diminished ability to deliver blood to sustain peripheral work and render the heart more susceptible to disease. The RV also undergoes remodeling with age, though these processes are unclear. In fact, unlike the LV, the changes in RV function with age are still contentious, with some groups reporting that RV systolic function is preserved (Kawut et al., 2011) while others suggesting that RV systolic function declines with advanced age (Kuo et al., 2018). The changes in RV diastolic function with age are also not clear, but one report suggests that RV diastolic function likely declines with age, as evidenced by larger contribution of atrial filling, suggesting altered compliance of the RV (Klein et al., 1999). Together, the physiological and anatomical changes that occur in the RV with age are not clearly described. Moreover, if functional systolic and diastolic differences underlie LV and RV aging, then molecular mechanisms are likely to differ between the ventricles as well. Therefore, we set out to understand the physiological and molecular mechanisms of RV and LV aging and for the first time, we report distinct pathophysiological and molecular aging mechanisms between the two ventricles.

## 2 MATERIALS AND METHODS

### 2.1 Animals

All experiments described in this study were conducted in accordance with institutional guidelines and approved by the Institutional Animal Care Users Committee of the University of Wyoming. Mice were housed 2–5 animals per cage in a temperature‐controlled room (21°C) on a standard 12/12 light/dark cycle. Food (Lab Diet 5001) and water were provided ad libitum. C57BL6 male and female mice were purchased from Jackson Laboratories (adult) or donated from the National Institutes of Aging Rodent Colony (aged). Adult (2–4 months), and aged (18–20 months) mice of both sexes were used. Animals were humanely euthanized with Fatal Plus (390 mg/mL pentobarbital sodium). The heart was dissected to separate the RV from the LV and septum. Ventricles were weighed, flash‐frozen, and stored at −80°C for subsequent analyses.

### 2.2 Right ventricle pressure volume loops

Mice were introduced to 3% isoflurane in 100% oxygen and maintained at 1.2 L/min while a PE‐50 tracheostomy tube was surgically placed. The ventilator was set to a stroke volume of 200–300 μL and stroke rate of 150 strokes/min. The thoracic cavity was opened exposing the apex of the heart and a small puncture made in the RV slightly superior to the apex. A pressure volume (PV) microcatheter (Millar PVR‐1030) was passed through the wound into the RV and positioned to optimize conductance signal. After catheter placement in the RV, the loops were briefly allowed to stabilize to ensure consistent baseline PV loops. The inferior vena cava just superior to the diaphragm was exposed and briefly occluded to reduce venous return to the heart to alter end diastolic volumes. A bolus of hypertonic saline (20% NaCl) was introduced through the jugular catheter to permit post hoc parallel conductance subtraction. The mouse was then heparinized prior to blood collection for volume cuvette calibration from the thoracic cavity. Lab Chart 8 Pro with PV‐Loop plug‐in was used to record the PV loops, perform saline and cuvette calibrations, and extract data.

### 2.3 Echocardiography

Cardiac function was assessed via transthoracic echocardiography using a Visual Sonics Vevo 2100 with a 40‐MHz probe (Fujifilm). Mice were induced with 2.0% isoflurane in 1.2 L/min 100% oxygen and maintained between 1% and 3% on an operative circuit nose cone titrated in 1.2 L/min 100% oxygen to keep heart rate between 400 and 600 BPM. Core temperature was monitored via a lubricated rectal probe and maintained at 37°C. Respiratory rate and heart rate were monitored with a 4‐lead limb ECG. Two‐dimensional parasternal long axis (LA) B‐Mode and parasternal short axis (SA) B‐Mode, and M‐Mode views of the LV were captured. Using VevoLab software, cine loop analysis was conducted on cardiac cycles between respirations. Endocardial borders were traced in LA B‐Mode up to the aortic valve to assess total area, volume, ejection fraction (EF) [EF = (LV end‐diastolic volume (LVEDV) − LV end systolic volume (LVESV))/LVEDV × 100], fractional shortening (FS) [FS = (LV internal diameter (LVIDd; diastole) − LVIDs; systole (LVIDs)/LVIDd × 100)], and cardiac output (CO). SA M‐mode tracings were captured to visualize the motion of the anterior and posterior wall to assess anterior and posterior LV wall thickness during diastole and systole. Right‐sided views: parasternal short axis views of the RV were captured in B‐mode and M‐mode. The PA was captured in B‐mode and pulse wave Doppler views of the PA were captured for PA acceleration times (PAT). RV M‐mode tracings were used to assess wall thickness during diastole and systole (RVFW;s and RVFW;d). Short axis B‐mode tracings were used to assess RV fractional area change (FAC). Calculations for stroke volume (SV), cardiac output (CO) and mean pulmonary artery pressure performed using from Vevo Lab software.

### 2.4 Histology

Whole hearts were removed, washed, and frozen in optimal cutting temperature buffer. Hearts were transversely cut into 6 μm sections cut using a pre‐cooled cryostat (Leica Biosystems). Hematoxylin and eosin staining was used to visualize cardiac structure following standard protocols. Briefly, sections were thawed at room temperature for 10 min then placed into Bouin's solution (picric acid) for perfusion fixation. Slides were then dehydrated using alcohol and vitrified in dimethylbenzene. Sections were then stained with hematoxylin and differentiated with 0.3% acid alcohol and washed in water. After washing, sections were covered in eosin for 30 s before washing with water until the tissue slide was clear. Sections were dipped in 70% ethanol five times, followed by five dips in 100% ethanol. Images were acquired at 4×.

### 2.5 RNA sequencing

LV and RV RNA was isolated and cleaned using commercially available kit with genomic DNA depletion (Qiagen RNeasy). For each sex,12 TruSeq RNA libraries (24 total) were sequenced using the NovaSEQ 6000 platform to generate 150‐bp paired‐end reads. Data were analyzed as previously described (Yusifov, Chhatre, Zumo, et al., 2021). The quality and contamination of the reads were evaluated using FastQC. The BDUK tool was used to trim the reads for adapter contamination, while Trimmomatic was used to remove reads with a minimum PHRED‐scaled quality score of 28. HISAT2 genome aligner (Kim et al., 2019) was used to align the read data with the mouse reference genome assembly version 38.97 and genome annotation version 38 from Ensembl. StringTie version 1.3.4 was utilized to assemble transcripts (Pertea et al., 2015), and EdgeR was used to analyze the raw counts of identified transcripts and genes for differential expression (Robinson et al., 2010). Counts were normalized, expression dispersion was estimated, and Fisher's exact test was applied to estimate statistical significance and determine the false discovery rate (FDR). Genes up‐ or downregulated at FDR <0.05 were further investigated for enrichment of specific expression pathways using Gene Set Enrichment Analysis (GSEA) and Kyoto Encyclopedia of Genes and Genomes (KEGG) analysis, conducted using the ClusterProfiler (version 3.14.3), ggplot2 (version 2_3.3.2), and enrichplot packages (version 1.6.1) in R software. The genome‐wide annotation was performed using the Bioconductor package (org.Mm.eg.db; version 3.10.0), and computing analysis was performed in the Advanced Research Computing Center at the University of Wyoming. The significance level for the GSEA and KEGG analyses was set at *p* < 0.05.

### 2.6 qRT‐PCR

To validate targets identified by RNAseq, we performed real‐time reverse transcription PCR. LV and RV RNA were reverse transcribed using standard procedures (iScript cDNA synthesis kit; Bio‐Rad). PCR was performed using iQ SYBR Green Supermix and normalized to the housekeeping gene β‐actin. Expression was calculated by ΔΔCt relative to adult within age and sex. Data are expressed as fold from sex‐matched adult within ventricle. Primer sequences were as follows. *β‐Actin* Forward: TGGACATCAGGAAGGACCTC, Reverse: ACATCTGCTGGAAGGTGGAC; *C1qB* Forward: CTCTGGGCTCTGGGAATCCA, Reverse: CCTCAGGGGCTTCCTGTGTA; *C3* Forward: AGCCCAACACCAGCTACATC, Reverse: GAATGCCCCAAGTTCTTCGC; *Acot1* Forward: GCAGCCACCCCGAGGTAAA, Reverse: GCCACGGAGCCATTGATG; *CD36* Forward: ATTGCGACATGATTAATGGCA, Reverse: GATGGACCTGCAAATGTCAGA; *Scd1* Forward: CTGACCTGAAAGCCGAGAAG, Reverse: AGAAGGTGCTAACGAACAGG.

### 2.7 Single‐cell suspension and flow cytometry

To characterize RV and LV aging immune cell populations, we performed flow cytometry analysis using BD FACS Melody Cell Sorter with three laser configurations (Violet: 405 nm, Blue: 488 nm, and YellowGreen: 561 nm). Adult and aged male mice were used for flow cytometry analysis. LV and RV cells were isolated and digested via standard procedures. Briefly, harvested hearts were minced and digested using collagenase II (Worthington, *LS004176*) at 37 degrees C for 1 h. Cells were triturated through 70 μm nylon sterile cell strainers (LifeScience Products, *CT‐229483*) and centrifuged at 400 rpm for 5 min. The supernatant was then collected and spun at 2000 rpm for 10 min to create an immune cell pellet. For normalization, spleen cells were also harvested using stain wash buffer with EDTA (SWBE) and mechanically disrupted through a 70 μm cell strainer, spleen cells were harvested using SWBE. Cell suspension concentrations were determined by 1:1 dilution of trypan blue (Gibco) to single cell suspension of RV, LV, and spleen using hemocytometer.

For extracellular/surface staining, single‐cell suspensions were prepared and plated (1 × 10^6^ cells/ 100 μL of 1 X PBS) followed by 30 min of incubation with Aqua Live/ dead dye (LIVE/ DEAD™ Fixable Aqua Dead Cell Stain Kit, Thermo Fisher Scientific, *L34957*) diluted in 1 X PBS for viability staining. Surface staining was performed using antibodies diluted in Stain wash buffer with EDTA (2% BCS in 1 X PBS and 0.01 M EDTA (SWBE)) according to manufacturer's recommendation for 30 min on ice under dark conditions with anti‐CD16/32 Fc‐shield (Tonbo BioSciences, *70‐0161‐M001*) blockade to reduce non‐specific staining. Antibodies used for surface staining were anti‐CD3‐FITC (BioLegend, clone‐ 17A2, *100204*), anti‐CD45‐PE (BioLegend, clone‐ 30‐F11, *103106*), anti‐CD11b‐PECyanine7 (BioLegend, clone‐M1/70, *101216*), anti‐GR1‐PerCP/Cyanine5.5 (BioLegend, clone‐ RB6‐8C5, *108428*) and anti‐IgG1‐PE (BioLegend, clone‐RTK2071, *400408*). Upon surface staining wells were washed with 1X SWBE. A BD Cytofix/Cytoperm™ Fixation/Permeabilization kit was used to fix stained cells according to the manufacturer's recommendation. Cells were fixed for 15 min with BD cytofix and then washed with 1X diluted Cytoperm™. Stained cells were then stored in −4°C for phenotypic analysis. Our gating strategy is demonstrated in Figure S1.

### 2.8 Statistical analyses

Morphometric and echocardiography data were analyzed by two‐way ANOVA (age × sex) with post hoc Student's *t*‐test to directly compare adult‐aged within sex and ventricle. Immune cell flow cytometry, hemodynamics, and gene expression data were analyzed via two‐way ANOVA (age × ventricle and age × sex, respectively) with post hoc Student's *t*‐test within ventricle.

## 3 RESULTS

**FIGURE 1:**
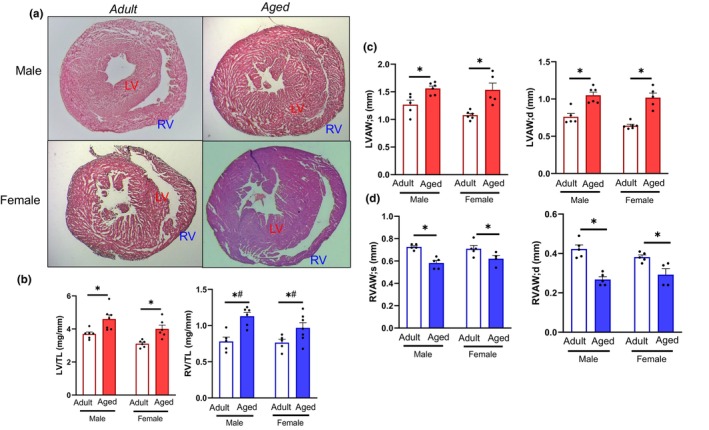
Morphometric remodeling of the RV and LV with aging. (a) A Histology of the adult and aged hearts demonstrate concentric hypertrophy of the LV and eccentric hypertrophy of the RV. (b) Cardiac mass normalized to tibia length (TL) demonstrates hypertrophy in both ventricles that is exacerbated in the RV with age compared to the LV (*, # Interaction: age × ventricle). (c) LV anterior wall thickness measured by echocardiography was thicker in aged animals during systole and diastole. (d) RV anterior wall thickness was thinner in aged mice during both systole and diastole. Red, LV; blue, RV; white bar, adult; closed bar, aged. Data were analyzed by three‐way ANOVA (age × sex × ventricle) with post hoc Student's *t*‐test to directly compare adult‐aged within sex and ventricle. *p < 0.05 by Student's *t*‐test within sex and ventricle. Values are presented as mean ± SEM; *n* = 5–7 mice/group.

Both ventricles underwent hypertrophic remodeling with age. While the LV underwent concentric hypertrophy, the RV dilated with advanced age (Figure 1a). We measured RV and LV mass and when normalized to tibia length to account for animal size, LV and RV mass were higher in aged animals compared to sex‐matched adults (Figure 1b). However, the increase in RV mass with age was higher compared to the increase in LV mass with age, as evidenced by a significant interaction age x ventricle. The type of hypertrophy differed with aging in each ventricle, with the LV undergoing concentric remodeling as evidenced by histology and echocardiography, where LV wall thickness during diastole and systole were higher in aged mice compared to adults (Figure 1c, Table 1). However, the RV thinned with advanced age during both systole and diastole, consistent with eccentric remodeling (Figure 1d, Table 1).

**TABLE 1:** Summary of echocardiography in the adult and aged right (RV) and left ventricle (LV).

	Male		Female	
	Adult	Aged	Adult	Aged
LV				
HR (bpm)	481 ± 33	477 ± 38	432 ± 12	404 ± 7
AAT (ms)	21.2 ± 8.4	16.6 ± 2.2	19.8 ± 4.8	**15 ± 3.5** *
LV wall, s (mm)	1.3 ± 0.2	**1.6 ± 0.1** *	1.1 ± 0.07	**1.5 ± 0.3** *
LV wall, d (mm)	0.76 ± 0.1	**1.1 ± 0.1** *	0.64 ± 0.04	**1.0 ± 0.13** *
LV Area, s (mm^2^)	4.1 ± 1.5	**6.0 ± 1.5** *	4.8 ± 0.7	**6.7 ± 1.9** *
LV Area, d (mm^2^)	10.1 ± 3.7	**13.5 ± 2.6** *	10.8 ± 0.6	**13.4 ± 1.9** *
IVRT (ms)	21 ± 2.7	20 ± 3	19.0 ± 3.8	31.0 ± 7.8
E/e’	26 ± 4.4	22 ± 4.8	20 ± 7	24 ± 12
EF (%)	69 ± 2.4	**62 ± 6.0** *	68 ± 2.6	64 ± 7.2
FS (%)	38 ± 4.6	**33 ± 4.1** *	37 ± 5.8	35 ± 12.4
SV (μL)	72 ± 23	56 ± 23	77 ± 24	79 ± 46
CO (mL/min)	34 ± 4	**25 ± 7** *	33 ± 4	32 ± 9
MAPSE (mm)	0.69 ± 0.04	**0.56 ± 0.06** *	0.69 ± 0.05	**0.55 ± 0.06** *
RV				
HR (bpm)	487 ± 89	461 ± 70	432 ± 42	402 ± 11
PAT (ms)	18.3 ± 3.2	20.5 ± 2.4	17.9 ± 2.1	18.5 ± 3.2
RV wall;s (mm)	0.73 ± 0.02	**0.58 ± 0.05** *	0.72 ± 0.06	**0.62 ± 0.06** *
RV wall;d (mm)	0.42 ± 0.05	**0.27 ± 0.03** *	0.38 ± 0.03	**0.29 ± 0.06** *
RV area;s (mm^2^)	3.6 ± 0.9	**5.7 ± 0.8** *	4.4 ± 1.2	**5.9 ± 1.0** *
RV area;d (mm^2^)	6.6 ± 2.0	**9.1 ± 0.8** *	8.1 ± 2.1	9.6 ± 1.4
E/A	0.64 ± 0.4	0.94 ± 0.8	0.47 ± 0.1	**0.92 ± 0.2** *
FAC (%)	46 ± 3.7	**35 ± 4.6** *	46 ± 2.3	**39 ± 1.9** *
SV (μL)	44 ± 13	37 ± 8	51 ± 13	35 ± 14
CO (mL/min)	22 ± 9	13 ± 7	22 ± 7	14 ± 6
TAPSE (mm)	0.70 ± 0.06	**0.53 ± 0.1** *	0.72 ± 0.1	**0.58 ± 0.1** *

*Note*: Data were analyzed via two‐way ANOVA (age × sex), followed by Student's *t*‐test. Data are presented as mean ± SEM; *n* = 5–7 mice/group. Bold: *p* < 0.05.

Abbreviations: AAT, aortic acceleration time; CO, cardiac output; d, diastole; E/A, ratio of peak velocity in early filling to atrial filling; E/e’, ratio of early diastolic mitral inflow velocity to early diastolic mitral annulus velocity; EF, ejection fraction; FAC, fractional area change; FS, fractional shortening; IVRT, isovolumic relaxation time; MAPSE, mitral annular plane systolic excursion; PAT, pulmonary acceleration time; SV, stroke volume; s, systole; TAPSE, tricuspid annular plane systolic excursion.

* *p* < 0.05 by Student's *t*‐test between age within sex and ventricle.

**FIGURE 2:**
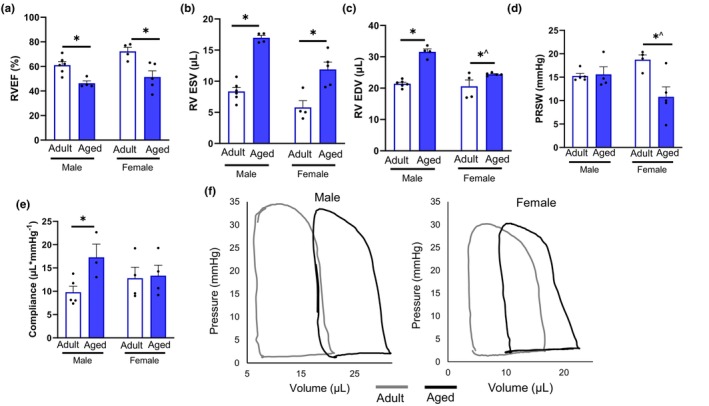
Analysis of RV function and hemodynamics by PV loops. (a) RV ejection fraction (EF) was lower in aged animals compared to sex‐matched adults. (b) RV end‐diastolic (EDV) and (c) end‐systolic volume (ESV) were higher in aged males and females although the increase in EDV with aging was more profound in male mice compared to female (*^ interaction age × sex). (d) Preload recruitable stroke work (PRSW) was lower in aged females (*^interaction age × sex). (e) RV compliance was elevated in aged males. (f) Representative male and female adult and aged RV PV loops. White bar, adult, closed bar, aged. Data were analyzed by two‐way ANOVA (age × sex), followed by Student's *t*‐test. *p < 0.05 by Student's *t*‐test within sex and ventricle. *^p < 0.05 interaction age × sex. Data are presented as mean ± SEM; *n* = 4–5 mice/group.

We then quantified RV and LV function by echocardiography (Table 1) and invasive hemodynamics (Table 2). RV fractional area change (FAC), a surrogate for ejection fraction, was reduced in both sexes with age (Table 1). Tricuspid annular plane systolic excursion (TAPSE) is a more sensitive measure of systolic function in the RV and was significantly lower with aging in both sexes (Table 1). To gain additional insight into the pathophysiology and hemodynamics of RV aging, we performed RV pressure‐volume (PV) loops (Table 2). RV EF was reduced in aged males and females (Figure 2a). RV end‐systolic volume (ESV) was higher in aged animals compared to sex‐matched controls (Figure 2b). End‐diastolic volume (EDV) was higher in both sexes but exacerbated in aged males (Figure 2c), confirming eccentric dilation. Preload recruitable stroke work (PRSW), an index of contractility, was lower in aged female mice compared to adults (Figure 2d). RV compliance was higher in aged males (Figure 2e). Together, both echocardiography and invasive hemodynamics  demonstrated impaired systolic dysfunction in the aging RV—a finding in contrast to the aging LV phenotype (top of Table 1) as described by others (Angelini et al., 2022).

**TABLE 2:** Summary of hemodynamics from pressure volume (PV) loops in the adult and aged RV.

	Male		Female	
	Adult	Aged	Adult	Aged
Ea (mmHg/mL)	2.2 ± 0.1	2.0 ± 0.2	2.1 ± 0.06	2.1 ± 0.3
RV EDV (μL)	21 ± 1	**32 ± 1** *	21 ± 1	**24 ± 0.2** *
RV ESV (μL)	8 ± 1	**17 ± 0.4** *	6 ± 1	**12 ± 1** *
PED (mmHg)	2.3 ± 0.3	2.3 ± 0.1	2.1 ± 0.2	2.8 ± 0.9
dP/dt max (mmHg/s)	1840 ± 169	2161 ± 275	2162 ± 55	**1568 ± 190** *
dP/dt min (mmHg/s)	−1799 ± 172	−2091 ± 259	−2055 ± 17	**−1535 ± 200** *
dV/dt max (μL/s)	941 ± 38	985 ± 170	713 ± 49	**1062 ± 142** *
dV/dt min (μL/s)	−689 ± 58	−707 ± 18	−645 ± 39	−834 ± 193
EDPVR	0.1 ± 0.01	**0.05 ± 0.01** *	0.09 ± 0.01	0.07 ± 0.01
CE	0.40 ± 0.03	0.52 ± 0.15	0.55 ± 0.05	0.36 ± 0.07
tau (ms); weiss	9.2 ± 0.9	7.1 ± 0.6	7.1 ± 0.16	9.9 ± 2.3
tau (ms); glantz	10.4 ± 0.9	**7.3 ± 0.8** *	7.5 ± 0.1	9.3 ± 1.0
Compliance	9.8 ± 1.3	**17.3 ± 2.5** *	12.8 ± 1.2	17.6 ± 4.6
PES (mmHg)	29.2 ± 1	29.0 ± 1	30.5 ± 1	**25.1 ± 1** *
RVSP (mmHg)	26 ± 1	26 ± 1	26 ± 1	**22 ± 1** *
ESPVR	1.7 ± 0.2	1.4 ± 0.3	2.5 ± 0.2	**1.2 ± 0.2** *
PRSW (mmHg)	15.3 ± 1	16.1 ± 2	18.7 ± 1	**10.8 ± 2** *
RV SW (mmHg/μL)	255 ± 30	275 ± 21	325 ± 10	**184 ± 27** *
RV EF (%)	61 ± 3	**46 ± 2** *	72 ± 2	**51 ± 5** *
RV SV (μL)	13 ± 1	15 ± 1	15 ± 1	13 ± 1
CO (mL/min)	6.5 ± 0.5	**8.3 ± 0.5** *	7.2 ± 0.4	6.9 ± 0.7

*Note*: Data were analyzed via two‐way ANOVA (age × sex), followed by Student's *t*‐test. Data are presented as mean ± SEM; *n* = 4–5 mice/group. Bold: *p* < 0.05.

Abbreviations: CO, cardiac output; CE, cardiac efficiency; dP/dt max, maximum rate of pressure change in the RV; dV/dt max, maximum rate of volume change in the RV; dP/dt min, minimum rate of pressure change in the RV; dV/dt min, minimum rate of volume of volume change, Ea, arterial elastance; EDPVR, end diastolic pressure‐volume relationship; ESPVR, end systolic pressure‐volume relationship; PED, end diastolic pressure; PES, end systolic pressure; PRSW, preload recruitable stroke work; RV SW, right ventricle stroke work; RVSP, right ventricle systolic pressure; SV, stroke volume; tau, isovolumic relaxation constant.

* *p* < 0.05 by Student's *t*‐test within sex.

**FIGURE 3:**
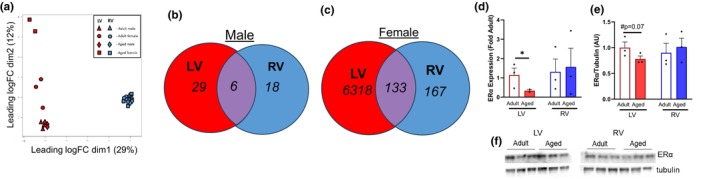
Differentially expressed genes (DEG) and estrogen receptor α (ERα) expression between adult and aged mice in the LV and RV. (a) Principal component analysis (PCA) plot showed distinct gene profiles in the LV and RV with aging. (b) Venn diagrams depicting the number of DEG between the ventricles with aging in males and (c) females. (d) Expression of ERα declines with age in the female LV but not RV, alongside (e) declines in protein expression of ERα. (f) Representative immunoblot. Red, LV; blue, RV, white bar, adult; closed bar, aged. Data was analyzed via Student's *t*‐test. *p < 0.05 by Student's *t*‐test within sex and ventricle. *n* = 3 mice/group. Data are presented as mean ± SEM.

To begin to elucidate the mechanisms of ventricle‐specific aging, we performed bulk RNAseq in RV and LV samples. As expected, the LV and RV were separated by principal component analysis (Figure 3a), suggesting distinct gene expression by ventricle, with the female LV changing more robustly than the RV. We compared differentially expressed genes (DEG) by age within the LV and RV. In male mice, only six genes (11%) were shared with aging in both ventricles (Figure 3b) and in female mice, 133 genes (2%) were shared with age (Figure 3c), suggesting distinct changes in gene expression with age in the LV and RV. The number of DEG in the female LV was exponentially larger than in the RV with aging, thus to identify potential mechanisms that may explain why gene expression in the female LV changed so robustly with age, we quantified estrogen receptor α (ERα) protein and gene expression. ERα gene expression was lower in the aging LV but not the aging RV (Figure 3d) compared to the adult, concomitant with lower protein expression only in the LV (Figure 3e,f). Neither gene nor protein expression of ERα changed with age in the male LV or RV (Figure S2).

**FIGURE 4:**
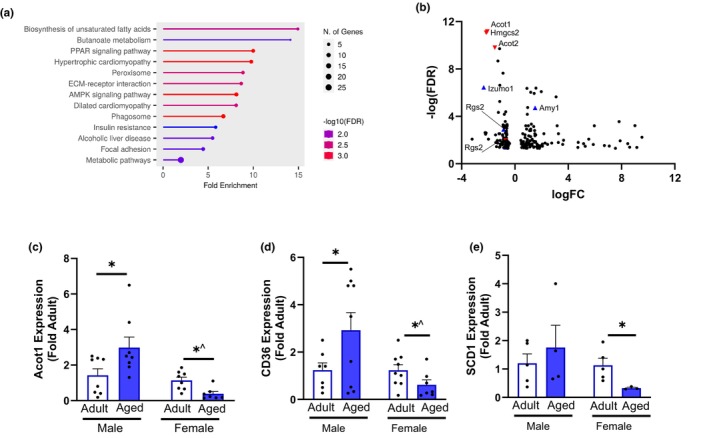
Unique RV aging DEGs. (a) Male and female RV aging KEGG analysis identified predominately metabolic pathways in the aging RV. (b) Volcano plot identifying DEG in the male and female RV. Red symbols represent top 3 female genes at FDR <0.01, blue symbols represent top 3 male genes at FDR <0.01. (c) Acot1 gene expression was lower in aged females but higher in aged males. (d) CD36 gene expression was lower in aged females but higher in aged males. (e) Scd1 expression was lower in aged females. White bar, adult; closed bar, aged. Data was analyzed by two‐way ANOVA (sex × age) followed by post hoc Student's *t*‐test. *p < 0.05 within sex and ventricle, *^interaction age × sex. *n* = 7 mice/group. Data are presented as mean ± SEM.

We first analyzed the DEG unique to the aging male and female RV and found many up‐ and downregulated targets (all RV DEG in Table S1). KEGG enrichment analysis identified metabolic pathways including biosynthesis of unsaturated fatty acids, PPAR signaling, and AMPK signaling **(**Figure 4a). We identified the three most robust DEGs by FDR for females (*Acot1, Hmgcs2*, and *Acot2*) and the two most robust in males (*Izumo1* and *Amy1*) (Figure 4b). The only shared RV‐specific gene was the regulator of G‐protein signaling 2 (*Rgs2*) which was downregulated in both the male and female‐aged RV compared to the adult. We confirmed the downregulation of Acot1 (acyl‐coA thioesterase 1) by PCR and found downregulation in the aged female but modest upregulation in the aged male R (Figure 4c). Based on the strong enrichment of lipid metabolism pathways, we validated the expression of a few key targets. Fatty acid transporter cluster of differentiation 36 (CD36) was downregulated in the aged female RV (Figure 4d) alongside downregulation of stearoyl‐CoA desaturase (Scd1; Figure 4e). Together, our analysis revealed dysregulation of metabolic pathways in the aging RV and identified novel RV‐specific mechanisms of aging.

**FIGURE 5:**
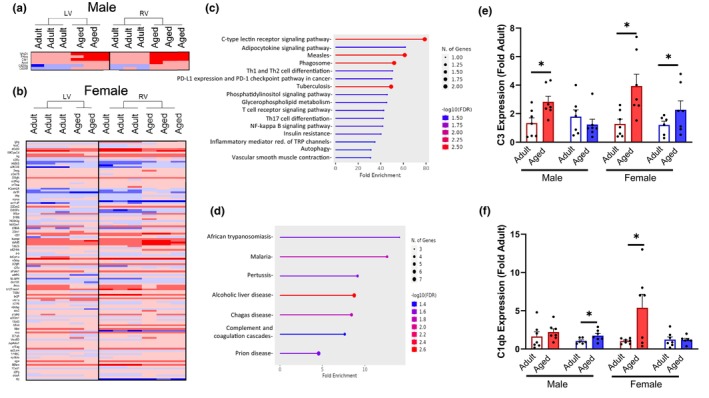
Shared aging RV and LV DEGs. Heat map demonstrating upregulation of inflammatory markers in the RV and LV with age in both (a) female and (b) male mice. (c) Male and (d) female‐shared aging KEGG analysis identified pathways associated with inflammation. (e) Main effect of age for C3 gene expression with upregulation in LV and RV compared to adults. (f) C1qb gene expression was higher in aged LV and RV (main effect age). Red, LV; blue, RV; white bar, adult; closed bar, aged. Data was analyzed by two‐way ANOVA (ventricle × age) followed by post hoc Student's *t*‐test. *p < 0.05 by Student's *t*‐test within sex and ventricle. *n* = 7 mice/group. Data are presented as mean ± SEM.

**FIGURE 6:**
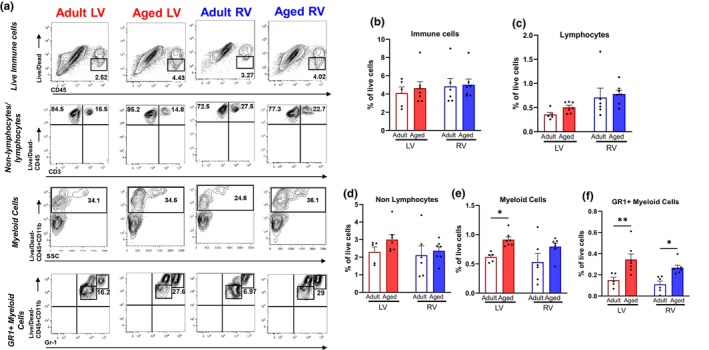
Analysis of RV and LV immune cell populations in adult and aged male mice using flow cytometry. (a) Representative contour plot of live immune cells (Lin‐CD45+), non‐lymphocytes (Lin‐CD45+), lymphocytes (Lin‐CD3 + CD45+), myeloid cells (Lin‐CD45 + CD11b+), and GR1+ myeloid cells (Lin‐CD45 + CD11b + Gr‐1+). Total percentages of (b) immune cells, (c) lymphocytes, (d) non‐lymphocytes, (e) myeloid cells, and (f) GR1+ myeloid cells after back calculation to total number of viable cells. Graphs show pooled data from two independent experiments (*n* = 3–5 mice/group). Red, LV; blue, RV; white bar, adult; closed bar, aged. Data were analyzed via two‐way ANOVA (age × ventricle), following by Student's *t*‐test. *p < 0.05, **p < 0.005 by Student's *t*‐test within sex and ventricle. Data were presented as mean ± SEM.

Successful therapy for the aging heart will likely require targeting of both ventricles, thus we analyzed genes that were differentially expressed in both the RV and LV with age. Both male (Figure 5a) and female (Figure 5b) hearts upregulated inflammatory pathways and genes consistent with inflammatory diseases (Figure 5c,d). We confirmed the expression of these shared targets and found that regulators of inflammation such as C3 and C1qb were upregulated with age in both ventricles (Figure 5g,h). Due to the shared genes implicating inflammation and immune pathways, we characterized RV and LV aging immune cell populations by flow cytometry. We focused our efforts on males due to shared inflammatory pathway enrichment across both sexes and due to the magnitude of inflammation pathway analysis hits in the males. No differences were detected in live immune cell populations (CD45^+^), non‐lymphocytes (CD3‐CD45+ ^−^), or lymphocyte (CD45 + CD3+) subpopulation percentages (Figure 6a–d). However, aging resulted in a higher percentage of myeloid‐derived cell populations (Lin‐CD45 + CD11b+) (Figure 6e). Myeloid derived cells (monocytes, macrophages, and neutrophils) were higher with aging (main effect of age) in both ventricles. Both the aged LV and RV also had higher percentages of GR+ myeloid‐derived cells (CD3‐CD45 + CD11b + Gr1+) (Figure 6f) compared to adult. Together our findings reveal concurrent upregulation of inflammatory pathways and immune cell populations in both ventricles in male and female hearts.

## 4 DISCUSSION

Age‐related adaptations to the myocardium are well described in the LV, whereas RV aging literature is limited and lacks consensus. Given the predictive power of RV function on clinical outcomes (Goliasch et al., 2015; Haddad et al., 2008), efforts to understand the mechanisms of RV aging are warranted. Here, we quantified functional and morphological differences between the ventricles with aging as well as identified shared and distinct transcriptomic mechanisms of aging. We show distinct hypertrophic phenotypes between the aged RV and LV concomitant with different functional outcomes. These differences also manifested transcriptionally, where the aged RV transcriptome enriched with metabolic and extracellular matrix pathways distinct from the LV. Together, we demonstrate that the RV and LV are unique cardiac chambers that age distinctly, suggesting current LV‐based approaches to target RV aging will likely not be fruitful.

The RV underwent dilation and eccentric hypertrophy with aging, concomitant with systolic dysfunction as measured by echocardiography and invasive hemodynamics. This RV aging phenotype is distinct from the concentric hypertrophy and diastolic dysfunction as well reported in the aged LV (Lakatta & Levy, 2003). Aging also increased RV EDV and ESV, indicative of a dilated aged ventricle that is not only holding more volume but also ejecting less blood. The importance of a dilated RV has recently been established by studies that have aimed to understand predictors of RV disease progression. Rischard et al. performed right heart catheterization and cardiac MRI on patients with pulmonary hypertension and found that a decrease in RV EDV was the best predictor of functional recovery outcomes (Rischard et al., 2023). In patients with arrhythmogenic right ventricular dysplasia cardiomyopathy, RV ventricular dilation was also a marker for disease progression during right heart failure (Peters, 1996). Both of these studies emphasize the importance of preventing excess RV chamber dilation to prevent right heart failure progression. The temporal changes in RV function with age still lack consensus. In a preclinical study using invasive hemodynamics in 80‐week‐old rats (comparable to ~55 years in humans), the authors found healthy aging increased RV wall thickness and contractility compared to 11‐week‐old adult controls (Sharifi Kia et al., 2021). A clinical study of similarly aged healthy adults (mean age 58) also suggested thickening of the RV outflow tract wall by echocardiography (Henein et al., 2014). However, other clinical reports demonstrate impaired systolic function measured by TAPSE‐pulmonary artery systolic pressure ratios in healthy cohorts ranging from age 20 to 80 (Wolsk et al., 2019). Thus, it seems possible that perhaps middle‐aged or early aging is associated with concentric remodeling and preserved (or enhanced) systolic function but that as aging continues progresses to dilation and systolic dysfunction. Future longitudinal studies or more comprehensive analyses of RV morphological and functional remodeling will be necessary to understand the trajectory of age‐associated changes in the RV.

We present the first RV‐specific transcriptional signature with aging that implicates metabolic dysfunction, specifically lipid metabolism. Given the paucity of RV datasets, it is difficult to discuss our transcriptomic signature in the context of others. However, in a model of accelerated aging in the mitochondrial DNA mutator mouse, Gorr et al. have shown enrichment of inflammation, fibrosis, and cardiomyopathy pathways in the RV (Gorr et al., 2022). The authors not only compared gene expression but also changes in protein abundance. They found that proteins that were upregulated post‐transcriptionally were associated with mitochondrial protein translation and electron transport chain assembly. Our KEGG analysis supports the enrichment of similar pathways, affecting genes in pathways such as lipid digestion and absorption, PPAR signaling, and other metabolic pathways. The most significant differentially expressed gene was Acot1, which reverts acyl‐CoAs back to free fatty acids (FFA). Acot1 plays a central role in providing balance in FFA concentrations and ultimately the rate of mitochondrial β‐oxidation. Work in young models of cardiac disease has demonstrated a protective role for Acot1 in cardiac remodeling and cardiac energy production (Xia et al., 2015). Peroxisome proliferator‐activated receptor alpha (PPARα), a transcription factor that modifies the expression of genes involved in fatty acid oxidation, is exquisitely regulated by acyl‐CoAs and FFAs (Dongol et al., 2007) and expression decreases with aging in the myocardium (Iemitsu et al., 2002). The ability of Acot1 to provide substrates for PPARα to affect fatty acid metabolism during cardiac aging is an unexplored field that may provide mechanistic insight into RV aging. Ultimately, the downregulation of Acot1 in the aging female RV would limit β‐oxidation potential and contribute to a shift in metabolism. Indeed, the aging heart undergoes a metabolic switch characterized by restricted metabolic flexibility and a decline in rates of fatty acid oxidation (Kates et al., 2003). As of yet, it is unclear if these mechanisms directly translate to the aging RV, as little is known about aged RV metabolism, particularly fatty acid utilization. Therefore, compensatory mechanisms to preserve or increase available energy to a metabolically impaired RV could be distinct from the aged LV. In the adult myocardium, recently published work demonstrates differences in metabolic substrate use between isolated adult RV and LV cardiomyocytes. The RV had a greater rate of fatty acid oxidation and utilization of fatty acids than the LV (Nguyen et al., 2024). If the RV relies on fatty acid use more than the LV, age‐associated mitochondrial dysfunction (Lesnefsky et al., 2016) and changes in substrate utilization could be more detrimental to the aged RV than the aged LV. Ongoing studies will aim to elucidate the role of Acot1 in mitochondrial and metabolic pathways unique to the aging RV.

Until ventricle‐specific targeted therapies become available, it is likely that we will need to treat cardiac aging by interventions that target both ventricles. Therefore, we also analyzed shared LV and RV DEG with aging. We found significant enrichment of inflammation pathways. Shared RV and LV DEG enriched in pathways including Th1, Th2, and Th17 cell differentiation pathways and T cell receptor signaling pathways. C‐type lectin receptor signaling pathway was the most enriched pathway, which we confirmed with the upregulation of C3 gene expression. We identified *Prkcq* (protein kinase C theta) among the top regulated genes in the male heart. *Prkcq* controls several aspects of T‐cell signaling (Yang & Miller, 1999) that likely become dysregulated with age. Aging also reduces function of hematopoietic stem cells, circulating naïve T cells, and results in an overall decline in adaptive and innate immune systems termed “immunosenescence” (Pawelec, 2018). Immunosenescence has been linked to early cardiovascular events (Cesari et al., 2003), further highlighting the importance of the immune system in promoting cardiac function. Aging also impacts overall immune cell populations, increasing total leukocytes (CD45^+^), neutrophils, and several monocyte subpopulations (Esfahani et al., 2021) in the whole heart. However, we do caution that these previous reports were either performed in isolated LV or whole heart homogenates and are likely skewed to favor the LV given that it composes the majority of ventricular mass. Gorr et al. have previously shown in adult male rats that the RV has more CD45+ immune cells and a larger percentage of macrophage and dendritic cells compared to the adult LV (Gorr et al., 2020). Although we show no differences between the ventricles in total percent viable immune cell populations, we do detect differences in some immune sub‐populations. Aging resulted in increased percentages of total myeloid‐derived cells in the LV and significantly higher percentages of GR1+ myeloid sub‐populations in both the aged RV and LV. While aged RV cellular phenotypes are still unclear, ongoing work aims to identify ventricle‐specific immune populations and their contributions to the cardiac aging phenotype.

The inclusion of both male and female mice is a strength of the current work. We (Yusifov, Chhatre, Koplin, et al., 2021) and others (Han et al., 2022) have previously reported that the LV undergoes distinct remodeling by sex. Our previous work identified that DEG in the aging female LV clustered in immune response pathways and ECM organization while in the male LV, KEGG analysis suggested enrichment of proteasome and oxidative phosphorylation (Yusifov, Chhatre, Koplin, et al., 2021). Here, we add to some of these previous observations by also demonstrating sex differences in RV aging. Although both male and female mice demonstrated impaired RV systolic function with age, there were subtle differences by sex, with males undergoing worse dilation and females demonstrating impaired contractility. Female mice expressed many more DEGs in the aging RV compared to aged male counterparts, sharing only one DEG with age. Female RV aging was also characterized by reduced expression of fatty acid metabolism genes, while these tended to be upregulated in the aging male. Interestingly, we also found that the transcriptional changes with age in the female LV were more robust than in the RV. Although speculative, perhaps the loss of estrogen with female aging has a larger impact on the LV than the RV. Indeed, a recent analysis of the Human Cardiac Cell Atlas compared RV and LV myocytes and found through upstream regulator analysis that β‐estradiol modulates gene expression in cardiomyocytes. The authors hypothesized that β‐estradiol genes are disproportionately important for the RV. Consistent with this notion, ER pathway targets were more frequently expressed in the RV than the LV (Prisco et al., 2022), and have been reported to decline with advanced age in whole heart tissue (Gurrala et al., 2021). Our findings support this observation, where we show that expression of ERα is lower during aging only in the LV, not the RV, suggesting the decline seen in previous reports is likely due to the much larger mass of the LV. Future mechanistic studies into how estrogen and estrogen signaling differentially impact the female LV and RV should provide important insight. Further insight into sex‐specific mechanisms of RV and LV aging differences is still required, but this hypothesis is exciting given the wide reports of sex differences in cardiac aging, RV function in pulmonary hypertension, and other clinical contexts. A focus on sex differences between the ventricles in cardiac aging will be warranted to permit identification of therapies to promote healthy aging for both aging men and women.

## 5 LIMITATIONS

We acknowledge several limitations. We acknowledge that only male mice were used to characterize immune cell populations. We chose this approach due to shared enrichment across both sexes of inflammatory pathways and due to the variety of KEGG pathway analysis hits in the males. However, the paucity of sex‐specific immune cell population aging data neccesitates further investigation. Mice in the present work were housed in Laramie, WY, located 7220 ft (2200 m) above sea level. Thus, adult control mice that were born and bred in our breeding colony likely adapt to our local mild hypoxic stress, whereas aged mice from NIA or commercial vendors may not have adequate time to adapt to the hypoxic stress. Therefore our findings may not directly translate to those conducted at sea level and future work should aim to understand how hypoxia plays a role in the aging right and left heart. Given the gap in understanding of mechanisms of RV aging, we focused our efforts on morphological, functional, and transcriptional changes that occur in the RV and how these may differ by sex. Sex differences in LV aging (Angelini et al., 2022; Kane et al., 2021; Sotomi et al., 2021) and transcriptional changes that underlie aging in the left side of the heart have been reported by us and others (Gerdes Gyuricza et al., 2022; Yusifov, Chhatre, Koplin, et al., 2021). Age‐related transcriptional changes are not necessarily mirrored in protein products (Gerdes Gyuricza et al., 2022). Due to the nature of bulk RNAseq, we are unable to determine from which cell types our differences emerged, however, we suspect that a multitude of cell types (fibroblasts, myocytes, and endothelial cells) display phenotypic and transcriptomic changes with age that may vary by ventricle. For example, a recent single‐cell RNAseq (scRNAseq) comparison of young and aged mouse whole hearts revealed that fibroblasts underwent the most significant changes in gene expression. Aged cardiac fibroblasts showed changes in the expression of inflammatory, extracellular matrix organization, angiogenesis and osteogenic genes (Vidal et al., 2019). The RV and LV differ significantly with respect to these cell types, though these differences remain poorly understood (Tucker et al., 2020) and have not yet been studied in the context of aging. The growing utilization of scRNAseq technologies will permit the teasing apart of some of these age‐related cell‐type specific changes and should yield valuable insight into new mechanisms of RV aging.

## 6 CONCLUSIONS

In conclusion, we aimed to understand pathophysiological and molecular mechanisms of RV aging and how they differ from those widely established in the LV. The aged RV presents an eccentric compliant and dilated phenotype associated with systolic dysfunction. Although the precise mechanisms driving this phenotype remain unclear, it may be influenced by changes in metabolic pathways such as fatty acids. Together, we strongly emphasize that the mechanisms of aging in the RV are distinct from those in the LV. Understanding these differences is critical to establishing ventricle‐specific therapies to improve healthy cardiac aging.

## AUTHOR CONTRIBUTIONS

*Conceptualization*: BDM, AY, JPG, DRB. *Methodology*: BDM, AY, JPT, CRH, TR, SBS. *Visualization*: TR, DRB. *Writing‐ original draft*: BDM, DRB. *Writing‐ reviewing & editing*: BDM, AY, JPT, CRH, SBS, TR, JPG, DRB.

## CONFLICT OF INTEREST STATEMENT

The authors declare no conflicts of interests.

## Supporting information


Data S1.

Data S2.

Data S3.

## Data Availability

The RNAseq data are publicly available in the NCBI database under BioProject PRJNA1128707. Other data is available from the corresponding author upon reasonable request.
