# Supplementary material for: Molecular and physiological mechanisms of aging are distinct in the cardiac right and left ventricles
Source: Aging Cell. 2024 Sep 19;24(1):e14339. doi: 10.1111/acel.14339 (PMC11709097; doi:10.1111/acel.14339)
Supplement: Supplementary file 1 — Data S1. [file ACEL-24-e14339-s003.pdf]

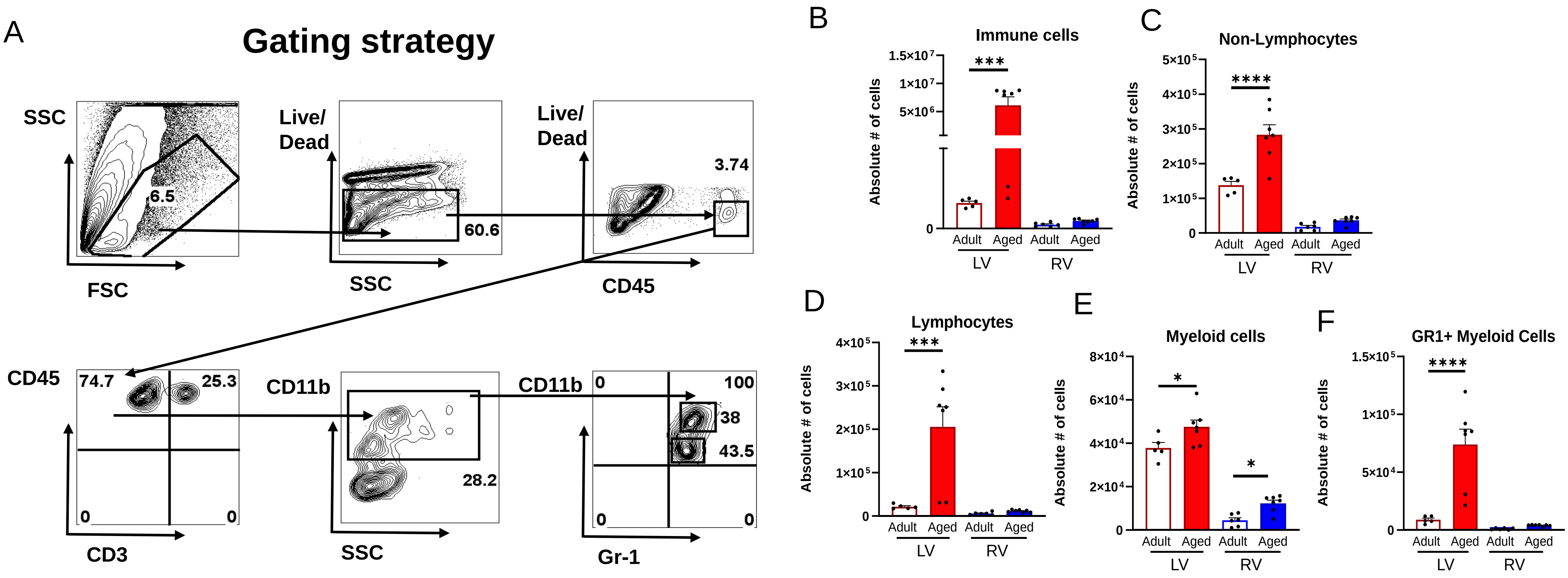

Supplemental Figure 1. RV and LV flow immune cell characterization gating strategy and absolute cell numbers. A) Representative contour plot progression of live immune cells (Live/Dead-CD45+), non-lymphocytes (Live/Dead-CD3-CD45+), lymphocytes (Live/Dead-CD3+CD45+), myeloid cells (Live/Dead-CD3-CD45+CD11b+) and inflammatory myeloid cells (Live/Dead-CD3-CD45+ CD11b+Gr-1+) depicting gating strategy used. Absolute number of cells in B) immune cells, C) non-lymphocytes, D) lymphocytes, E) myeloid cells and F) GR1+ myeloid cell populations. Graphs show pooled data from two independent experiment (3-5 mice/group/ventricle). Red = LV, blue = RV, white bar = adult, closed bar = aged. *$p<0.05$,***$p<0.0005$,*****$p<0.00005$ by Student's t-test within sex and ventricle. Data are presented as mean ± SEM.

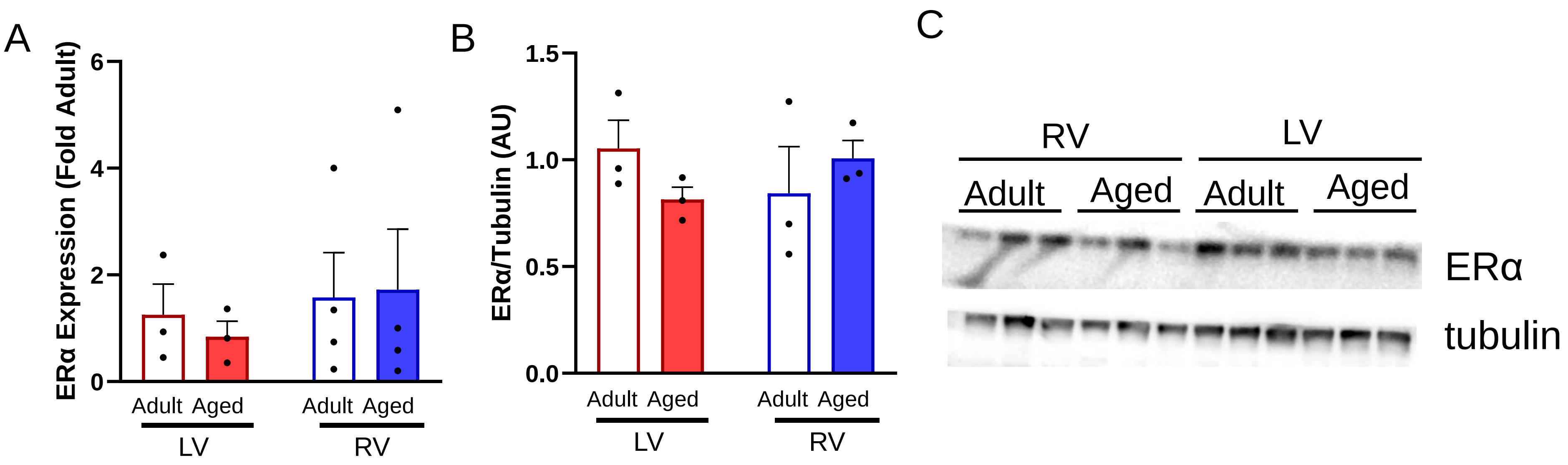

Supplemental Figure 2. Expression of ERα is unchanged in the male RV and LV with aging. A) mRNA and B) protein expression with C) representative immunoblot. Red = LV, blue = RV, white bar = adult, closed bar = aged. Data was analyzed via Student's t-test. n = 3-4 mice/group. Data are presented as mean ± SEM.
