# Supplementary material for: Molecular and physiological mechanisms of aging are distinct in the cardiac right and left ventricles
Source: Aging Cell. 2024 Sep 19;24(1):e14339. doi: 10.1111/acel.14339 (PMC11709097; doi:10.1111/acel.14339)
Supplement: Supplementary file 2 — Data S2. [file ACEL-24-e14339-s001.docx]

**Supplemental Table.** Differentially expressed genes (DEG) that change with age in the male and female RV. Shared DEG in the LV and RV with age in the male and female RV.
